# Motivation to Quit Tobacco and Its Determinants Among Adult Tobacco Users: A Primary Health Center-Based Cross-Sectional Study From Dehradun District, Uttarakhand, India

**DOI:** 10.7759/cureus.115905

**Published:** 2026-09-07

**Authors:** Basavaraj SY, Kanchan Gupta, Gargi Pandey, Joen Mathew, Vartika Saxena, Aswani Seth, Nilanjan Roy, Aakash Sachdeva, Mahendra Gehlot

**Affiliations:** 1 Community Medicine, All India Institute of Medical Sciences Rishikesh, Rishikesh, IND

**Keywords:** motivation to quit, primary healthcare, richmond scale, rural india, tobacco cessation, tobacco use

## Abstract

Background

Motivation to quit tobacco is a key determinant of cessation behavior and successful abstinence. However, evidence regarding factors influencing motivation to quit among rural tobacco users in India remains limited.

Objectives

The objectives of this study are to assess the level of motivation to quit tobacco and identify factors associated with motivation to quit among tobacco users attending a rural primary health center (PHC) in Uttarakhand and to assess the correlation between the nicotine dependence score and motivation to quit.

Methods

The current analysis is a secondary analysis of information gathered as part of a cross-sectional study evaluating tobacco users' reliance on tobacco. Between February and May 2026, adult tobacco smokers who visited Primary Health Center Raiwala, Uttarakhand, participated in a cross-sectional analytical study. A standardized questionnaire that assessed clinical profile, tobacco use history, and sociodemographic characteristics was used to collect data. The Richmond Scale was used to measure motivation for tobacco cessation. To identify predictors of motivation to stop smoking, a logistic regression analysis was conducted.

Results

Overall, 80 (30.0%; 95% confidence interval {CI}: 24.5-35.8) participants had moderate/high motivation to quit tobacco, while 187 (70.0%) had low motivation. Bivariate analysis showed significant associations with age, education, occupation, socioeconomic status, diabetes, age at initiation, the duration of tobacco use, and nicotine dependence (p<0.05). Multivariable logistic regression identified skilled/sales occupation (adjusted odds ratio {AOR}=2.66; 95% CI: 1.05-6.70), tobacco use for 10-20 years (AOR=0.25; 95% CI: 0.08-0.77) and >20 years (AOR=0.13; 95% CI: 0.03-0.52), and moderate/high nicotine dependence (AOR=0.38; 95% CI: 0.19-0.77) as significant predictors. Motivation was weakly and negatively correlated with nicotine dependence.

Conclusion

Approximately one-third of tobacco smokers in rural areas showed moderate to high motivation to stop. Lower motivation was linked to longer tobacco use duration and increased nicotine dependency, underscoring the need for focused cessation therapies at the primary healthcare level.

## Introduction

Tobacco use is one of the most significant public health challenges worldwide and is responsible for more than eight million deaths each year [1]. Nearly 80% of the world's tobacco users live in low- and middle-income countries, where the burden of tobacco-related disease continues to rise [1].

India is the second-largest tobacco consumer in the world, with 28.6% of adults using smokeless or smoked tobacco, according to the Global Adult Tobacco Survey (GATS-2) [2]. Tobacco use continues to be a significant cause of avoidable morbidity and mortality, with both smokeless and smoked tobacco products carrying a significant burden. The prevalence of tobacco use is especially high in rural areas, where continued tobacco use is a result of poorer socioeconomic level, less education, cultural acceptance, and restricted access to quitting programs. In India, socioeconomic disparities have also had a major impact on tobacco use patterns, with underprivileged groups bearing a disproportionately greater tobacco use burden [3]. Compulsive tobacco use despite being aware of its harmful effects is the main characteristic of nicotine dependence, a chronic neurobehavioral disorder. Tolerance, withdrawal symptoms, and enduring cravings are brought on by repeated nicotine exposure, which encourages tobacco use [4]. The necessity for specialized tobacco cessation interventions in high-risk populations is highlighted by the fact that people with higher levels of nicotine dependency are less likely to successfully quit, have more withdrawal symptoms, and are more likely to relapse [5].

One of the most significant behavioral factors affecting tobacco quitting and long-term abstinence is motivation to stop. It is a crucial indicator of effective cessation efforts and shows a person's readiness and motivation to start changing their habit. While people with low motivation frequently continue to use tobacco despite being aware of its detrimental health effects, those with higher levels of motivation are more likely to seek professional assistance, stick with cessation counseling, and make repeated attempts to quit [6]. Since motivation is dynamic and can change over time, assessing it provides useful information for developing customized quitting plans. Healthcare professionals can identify patients who need intensive counseling and behavioral interventions to improve preparedness to quit by routinely assessing motivation. The Richmond Test is a brief and validated instrument developed for use in primary care and clinical settings to assess motivation to quit tobacco. It has been widely used to predict the likelihood of cessation success and to guide the intensity of tobacco cessation interventions according to an individual's level of motivation [7].

Primary health centers (PHCs) represent the first level of contact between communities and the healthcare system in rural India. Under the National Programme for Prevention and Control of Non-Communicable Diseases (NP-NCD), PHCs are expected to deliver tobacco cessation interventions as part of routine care [8]. However, evidence regarding motivation to quit tobacco among rural populations attending PHCs in Uttarakhand remains limited.

Therefore, the present study aimed to assess the level of motivation to quit tobacco and identify factors associated with motivation to quit among tobacco users attending a rural PHC in Raiwala, Uttarakhand.

## Materials and methods

Study design and setting

The cross-sectional study was conducted at the Primary Health Center (Center for Rural Health, India Institute of Medical Sciences {AIIMS} {CRHA}) Raiwala, operating under the Department of Community Medicine of AIIMS Rishikesh, Uttarakhand. The present analysis represents a secondary analysis of data collected as part of a cross-sectional study assessing tobacco dependence among tobacco users.

Study duration

The study was conducted between February 2026 and May 2026 after obtaining ethical approval from the Institutional Ethics Committee (IEC) of AIIMS Rishikesh.

Study population

Adult tobacco users attending PHC Raiwala during the study period were included.

Inclusion and exclusion criteria

Adults over the age of 18 who were currently using smokeless or smoked tobacco, had consumed tobacco over the previous 30 days, and gave written informed consent were included in the study. The study excluded participants who declined to participate, were pregnant, or were extremely ill.

Sample size calculation

The sample size for the parent study was calculated to estimate the prevalence of nicotine dependence among tobacco users using the formula n=Z²pq/d². Assuming a prevalence of 78% moderate-to-high nicotine dependence from a previous study by Chahar et al., with a 95% confidence level and 5% precision, the calculated sample size was 264 participants [9]. Motivation to quit tobacco was a prespecified secondary objective of the parent study and therefore did not require a separate sample size calculation. The sample of 267 tobacco users enrolled to address the primary objective (the prevalence of nicotine dependence) was also used for the present analysis.

Sample adequacy was further supported using three complementary approaches. First, a post hoc power analysis performed in G*Power 3.1 (α=0.05, one-tailed, odds ratio {OR}=0.13 {significant and least OR from the adjusted regression model}, outcome prevalence=30.0%, and n=267) demonstrated an achieved statistical power of >80% (1-β=1.00), indicating sufficient power to detect the observed association. Second, the prevalence estimate of moderate/high motivation to quit tobacco (30.0%) had a 95% confidence interval (CI) of 24.5%-35.5%, indicating acceptable precision. Third, the final multivariable logistic regression included approximately 80 outcome events, providing an events per variable (EPV) of >10 (seven variables included in the final model), satisfying the recommended criterion for stable and reliable logistic regression estimates.

Sampling technique

The sampling strategy employed was consecutive sampling. Until the necessary sample size was reached, all qualified tobacco users who visited PHC Raiwala during the study period were recruited.

Data collection tools

Data were collected through face-to-face interviews in a private consulting room by trained interviewers using a pretested, structured questionnaire consisting of the following: (1) sociodemographic variables; (2) tobacco use history; (3) clinical profile; (4) Richmond Scale of motivation to quit tobacco with the categories ≤5=low motivation, 6-7=moderate motivation, and ≥8=high motivation [6]; and (5) Fagerström nicotine dependence scale for smokers (FTND) [10] and Fagerström nicotine dependence scale for smokeless tobacco (FTND-ST) users [11]. For significant (moderate-to-high) nicotine dependence, FTND≥5 (smokers), or FTND-ST≥5 (smokeless users) [10,11]. The dataset was complete, with no missing values for any study variables; therefore, no imputation or other missing-data handling procedures were required. In dual tobacco users, we used the one with a higher nicotine dependence score for calculating overall nicotine dependence.

Participants received brief tobacco cessation counseling using the 5As approach, a recommended evidence-based intervention for tobacco cessation in primary care settings [12].

Operational definitions

Current tobacco user is defined as any use of smoked or smokeless tobacco in the past 30 days [2]. Smoker is defined as the current use of any smoked tobacco product (bidi, cigarette, and hookah) [2]. Smokeless tobacco user is defined as the current use of any smokeless product (khaini, gutkha, zarda, pan masala with tobacco, etc.) [2]. Dual user is defined as the current use of both smoked and smokeless forms [2]. Socioeconomic status was classified using the modified BG Prasad 2026 scale with the latest All-India Consumer Price Index update; participants were categorized into the standard five classes (upper, upper-middle, middle, lower-middle, and lower) [13].

Data analysis

Data were analyzed using Jamovi statistical software v2.6 [14]. Descriptive statistics were used to summarize participant characteristics. Categorical variables were described as frequencies and percentages; continuous variables were described as mean (SD) or median (IQR), depending on normality as assessed by the Shapiro-Wilk test/Q-Q plot. Logistic regression analysis was used to identify predictors of motivation to quit tobacco. Correlation between motivation to quit scores and nicotine dependence scores was assessed using Spearman's correlation. A p-value of <0.05 was considered statistically significant. Variables with p<0.20 on univariable analysis, together with age, sex, education, and the type of tobacco use selected a priori, were considered for inclusion in the multivariable binary logistic regression model. The final model was specified using the original analysis strategy, and variables excluded from the final model were documented accordingly.

Ethics

Ethical approval was obtained from the Institutional Ethics Committee of AIIMS Rishikesh (reference number AIIMS/IEC/26/61, dated 13 February 2026). Written informed consent was obtained from all participants in either Hindi or English. Throughout the investigation, confidentiality was upheld, and information was stored in a password-protected file accessible only to the researchers.

## Results

The study comprised 267 current tobacco users who were enrolled at PHC Raiwala. Of the participants, 46.1% were over 60, 61.4% were men, and 74.9% were from a lower socioeconomic class. Of the sample, 43.1% were smokers, 34.5% used smokeless tobacco, and 22.5% reported using two tobacco products. Of the patients, 69.3% had moderate-to-high nicotine dependency, and 40.1% had attempted to quit at least once before (Table 1).

**Table 1:** Association of participant characteristics with moderate/high motivation to quit tobacco (N=267) *Statistically significant association at p<0.05

Variable	Number (%)	Moderate/High Motivation, n (%)	Low Motivation, n (%)	χ²	P-value
Age group				15.10	<0.001*
<45 years	52 (19.5)	25 (48.1)	27 (51.9)		
45-59 years	92 (34.5)	31 (33.7)	61 (66.3)		
≥60 years	123 (46.1)	24 (19.5)	99 (80.5)		
Sex				1.12	0.29
Female	103 (38.6)	27 (26.2)	76 (73.8)		
Male	164 (61.4)	53 (32.3)	111 (67.7)		
Education				7.75	0.021*
Primary or below	79 (29.6)	17 (21.5)	62 (78.5)		
Secondary	136 (50.9)	40 (29.4)	96 (70.6)		
Higher secondary and above	52 (19.5)	23 (44.2)	29 (55.8)		
Occupation				13.50	0.009*
Unemployed	123 (46.1)	26 (21.1)	97 (78.9)		
Professionals and technical workers	26 (9.7)	9 (34.6)	17 (65.4)		
Skilled/sales workers	57 (21.3)	27 (47.4)	30 (52.6)		
Clerical and service workers	44 (16.5)	14 (31.8)	30 (68.2)		
Agricultural/managerial/machine operators	17 (6.4)	4 (23.5)	13 (76.5)		
Socioeconomic status				11.50	0.003*
Lower class	200 (74.9)	49 (24.5)	151 (75.5)		
Middle class	46 (17.2)	22 (47.8)	24 (52.2)		
Upper class	21 (7.9)	9 (42.9)	12 (57.1)		
T2DM				3.93	0.048*
No	210 (78.7)	69 (32.9)	141 (67.1)		
Yes	57 (21.3)	11 (19.3)	46 (80.7)		
Hypertension (HTN)				1.0	0.794
No	194 (72.7)	59 (30.4)	135 (69.6)		
Yes	73 (27.3)	21 (28.8)	52 (71.2)		
Presence of comorbidity				1.30	0.250
No	146 (54.7)	48 (32.9)	98 (67.1)		
Yes	121 (45.3)	32 (26.4)	89 (73.6)		
Age at initiation of tobacco use				6.22	0.013*
<30 years	100 (37.5)	39 (39.0)	61 (61.0)		
≥30 years	167 (62.5)	41 (24.6)	126 (75.4)		
Duration of tobacco use				24.20	<0.001*
<10 years	31 (11.6)	20 (64.5)	11 (35.5)		
10-20 years	83 (31.1)	28 (33.7)	55 (66.3)		
>20 years	143 (53.6)	32 (20.9)	121 (79.1)		
Overall nicotine dependence				9.13	0.003*
Low	82 (30.7)	35 (42.7)	47 (57.3)		
Moderate/high	185 (69.3)	45 (24.3)	140 (75.7)		
Previous quit attempts				1.15	0.300
No	160 (59.9)	44 (27.5)	116 (72.5)		
Yes	107 (40.1)	36 (33.6)	71 (66.4)		
Type of tobacco used				2.83	0.240
Both smoking and smokeless	60 (22.5)	13 (21.7)	47 (78.3)		
Smoking only	115 (43.1)	39 (33.9)	76 (66.1)		
Smokeless only	92 (34.5)	28 (30.4)	64 (69.6)		
Family type				1.87	0.400
Three-generation family	73 (27.3)	21 (28.8)	52 (71.2)		
Joint family	93 (34.8)	24 (25.8)	69 (74.2)		
Nuclear family	101 (37.8)	35 (34.7)	66 (65.3)		
Marital status				2.80	0.250
Widow/divorced	33 (12.4)	6 (18.2)	27 (81.8)		
Married	221 (82.8)	69 (31.2)	152 (68.8)		
Unmarried	13 (4.9)	5 (38.5)	8 (61.5)		
Alcohol use				0.07	0.800
No	194 (72.7)	59 (30.4)	135 (69.6)		
Yes	73 (27.3)	21 (28.8)	52 (71.2)		

Motivation to quit among the participants

Overall, 70.0% of participants demonstrated low motivation to quit tobacco, whereas 12.7% and 17.2% exhibited moderate and high motivation, respectively. When moderate and high motivation categories were combined, only 30.0% (95% CI: 24.5-35.8) of participants reported moderate/high motivation to quit (Figure 1).

**Figure 1:**
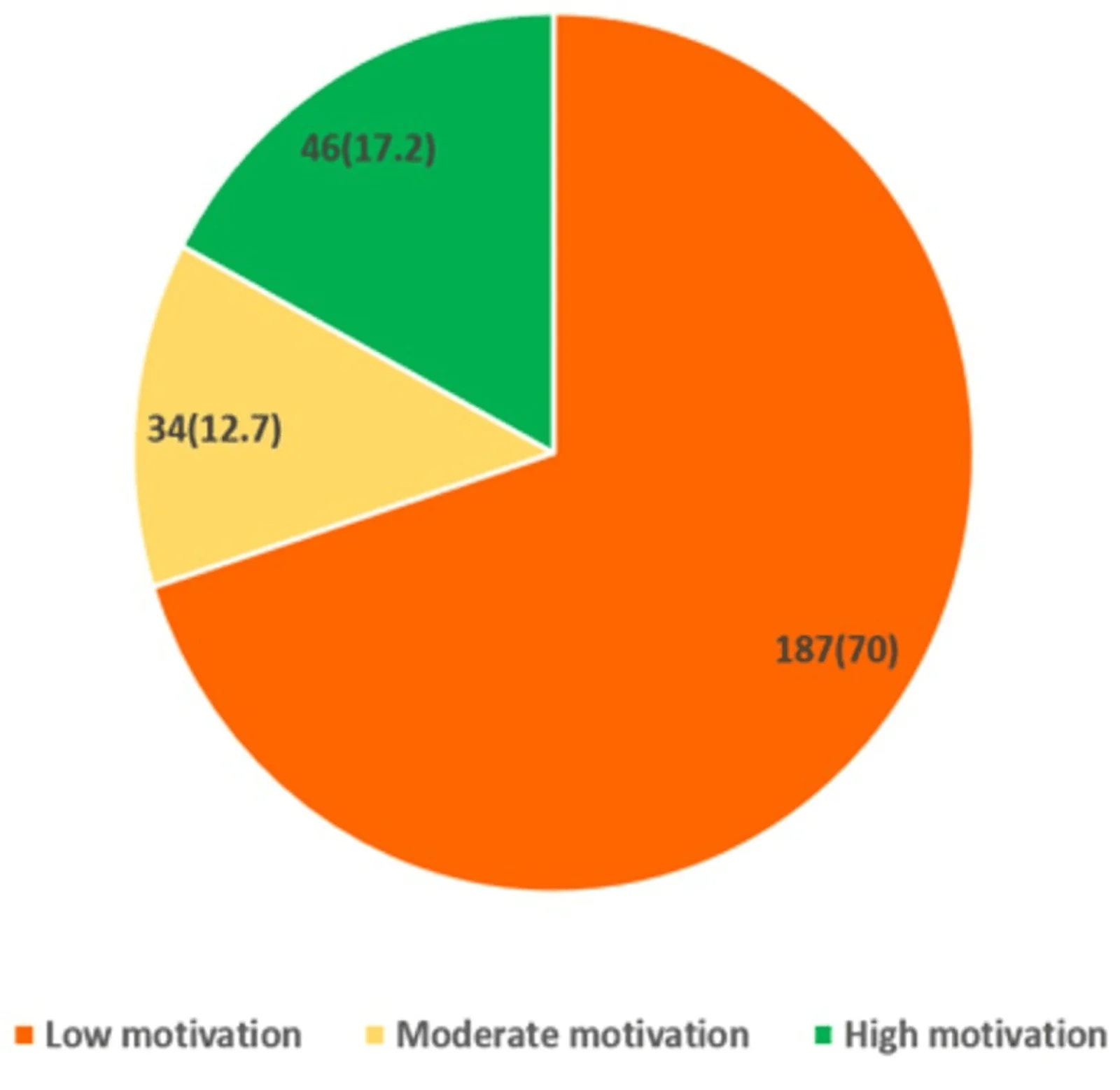
Frequency distribution of motivation to quit tobacco (N=267)

Among dual tobacco users (n=60), only eight (13.3%) had moderate motivation, and five (8.3%) had high motivation to quit. Among smoking-only users (n=115), 76 (66.1%), 15 (13.0%), and 24 (20.9%) showed low, moderate, and high motivation, respectively. Among smokeless tobacco users (n=92), only 28 (30.4%) had moderate-to-high motivation to quit. High motivation was most common among smoking-only users and least common among dual users (Figure 2).

**Figure 2:**
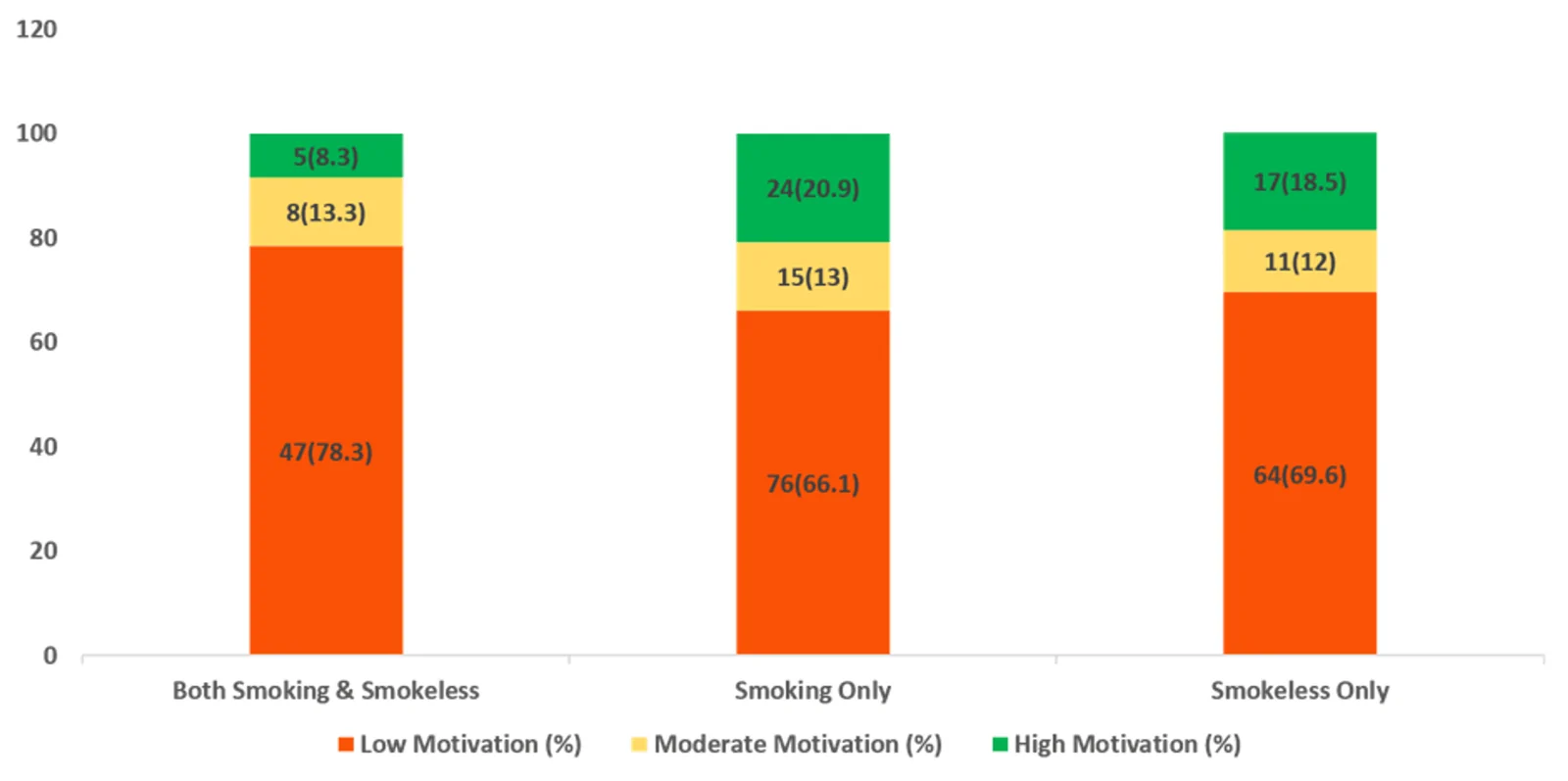
Motivation to quit based on the type of tobacco use (N=267)

Bivariate analysis for factors associated with moderate/high motivation to quit

Bivariate analysis showed that motivation to quit was significantly associated with age group (p<0.001), education level (p=0.021), occupation (p=0.009), socioeconomic status (p=0.003), type 2 diabetes mellitus (p=0.048), age at the initiation of tobacco use (p=0.013), the duration of tobacco use (p<0.001), and nicotine dependence status (p=0.003). Participants aged <45 years and those with higher education, shorter duration of tobacco use, and lower nicotine dependence demonstrated higher levels of motivation to quit (Table 1).

Predictors of moderate/high motivation to quit tobacco

Univariate logistic regression showed significant associations of age, higher education, skilled/sales occupation, middle socioeconomic status, age at tobacco initiation, the duration of tobacco use, and nicotine dependence with motivation to quit. Occupation, the duration of tobacco use, and nicotine dependence were found to be independent predictors of moderate/high motivation to stop using multivariable logistic regression. Diabetes mellitus was associated with significant nicotine dependence in univariable analysis (p=0.048). Its exclusion from the final adjusted model was based on the prespecified model selection procedure, as detailed in the Materials & Methods section. The odds of moderate/high motivation were substantially greater for skilled/sales workers than for jobless participants (AOR=2.66; 95% CI: 1.05-6.70; p=0.038). Participants with 10-20 years (AOR=0.25; 95% CI: 0.08-0.77; p=0.016) and >20 years (AOR=0.13; 95% CI: 0.03-0.52; p=0.004) of tobacco use and those with moderate/high nicotine dependence (AOR=0.38; 95% CI: 0.19-0.77; p=0.008) had significantly lower odds of moderate/high motivation. No independent associations were observed for age, education, socioeconomic status, age at initiation, previous quit attempts, or the type of tobacco use.

Model Performance

The final multivariable logistic regression model showed good overall fit (model χ²=45.9; degrees of freedom {df}=17; p<0.001), explaining 22.4% of the variance in motivation to quit (Nagelkerke R²=0.224). No evidence of multicollinearity was observed (variance inflation factor, 1.15-1.54; tolerance, 0.65-0.87) (Table 2).

**Table 2:** Logistic regression analysis for predictors of moderate/high motivation to quit tobacco *Statistically significant association at p<0.05 OR, odds ratio; CI, confidence interval

Predictor	Moderate/High Motivation, n (%)	Low Motivation, n (%)	Unadjusted OR (95% CI)	p	Adjusted OR (95% CI); p
Age group					
<45 years	25 (48.1)	27 (51.9)	Reference		Reference
45-59 years	31 (33.7)	61 (66.3)	0.55 (0.27-1.10)	0.090	1.61 (0.47-5.46); p=0.50
≥60 years	24 (19.5)	99 (80.5)	0.26 (0.13-0.53)	<0.001	1.74 (0.36-8.34); p=0.50
Sex					
Female	27 (26.2)	76 (73.8)	Reference		-
Male	53 (32.3)	111 (67.7)	1.34 (0.78-2.32)	0.290	-
Education					
Primary or below	17 (21.5)	62 (78.5)	Reference		Reference
Secondary	40 (29.4)	96 (70.6)	1.52 (0.79-2.91)	0.210	0.89 (0.39-2.02); p=0.80
Higher secondary and above	23 (44.2)	29 (55.8)	2.89 (1.34-6.23)	0.007	0.60 (0.19-1.92); p=0.40
Occupation category					
Unemployed	26 (21.1)	97 (78.9)	Reference		Reference
Professionals and technical workers	9 (34.6)	17 (65.4)	1.98 (0.79-4.94)	0.150	1.09 (0.29-4.12); p=0.90
Skilled/sales workers	27 (47.4)	30 (52.6)	3.36 (1.71-6.61)	<0.001	2.66 (1.05-6.70); p=0.038*
Clerical and service workers	14 (31.8)	30 (68.2)	1.74 (0.81-3.75)	0.161	1.58 (0.63-3.96); p=0.33
Agricultural/managerial/machine operators	4 (23.5)	13 (76.5)	1.15 (0.35-3.82)	0.821	0.90 (0.23-3.47); p=0.90
Socioeconomic status					
Lower class	49 (24.5)	151 (75.5)	Reference		Reference
Middle class	22 (47.8)	24 (52.2)	2.83 (1.46-5.48)	0.002	2.01 (0.84-4.80); p=0.12
Upper class	9 (42.9)	12 (57.1)	2.31 (0.92-5.81)	0.080	1.74 (0.44-6.98); p=0.43
Family type					
Nuclear family	35 (34.7)	66 (65.3)	Reference		-
Joint family	24 (25.8)	69 (74.2)	0.66 (0.35-1.22)	0.180	-
Three-generation family	21 (28.8)	52 (71.2)	0.76 (0.40-1.46)	0.410	-
Marital status					
Unmarried/widow/widower	11 (23.9)	35 (76.1)	Reference		-
Married	69 (31.2)	152 (68.8)	1.44 (0.69-3.01)	0.331	-
Diabetes mellitus (type 2)					
No	69 (32.9)	141 (67.1)	Reference		-
Yes	11 (19.3)	46 (80.7)	0.49 (0.24-1.00)	0.050	-
Hypertension					
No	59 (30.4)	135 (69.6)	Reference		-
Yes	21 (28.8)	52 (71.2)	0.92 (0.51-1.67)	0.801	-
Presence of comorbidity					
No	48 (32.9)	98 (67.1)	Reference		-
Yes	32 (26.4)	89 (73.6)	0.73 (0.43-1.25)	0.251	-
Age at the initiation of tobacco use					
<30 years	39 (39.0)	61 (61.0)	Reference		Reference
≥30 years	41 (24.6)	126 (75.4)	0.51 (0.30-0.87)	0.011	0.64 (0.30-1.35); p=0.24
Duration of tobacco use					
<10 years	20 (64.5)	11 (35.5)	Reference		Reference
10-20 years	28 (33.7)	55 (66.3)	0.28 (0.12-0.67)	0.004	0.25 (0.08-0.77); p=0.016*
>20 years	32 (20.9)	121 (79.1)	0.15 (0.06-0.33)	<0.001	0.13 (0.03-0.52); p=0.004*
Overall nicotine dependence					
Low	35 (42.7)	47 (57.3)	Reference		Reference
Moderate/high	45 (24.3)	140 (75.7)	0.43 (0.25-0.75)	0.003	0.38 (0.19-0.77); p=0.008*
Previous quit attempts					
No	44 (27.5)	116 (72.5)	Reference		Reference
Yes	36 (33.6)	71 (66.4)	1.34 (0.79-2.27)	0.281	1.26 (0.68-2.33); p=0.50
Type of tobacco used					
Smoking only	39 (33.9)	76 (66.1)	Reference		Reference
Both smoking and smokeless	13 (21.7)	47 (78.3)	0.54 (0.26-1.11)	0.091	0.99 (0.39-2.50); p=0.10
Smokeless only	28 (30.4)	64 (69.6)	0.85 (0.47-1.54)	0.602	1.73 (0.77-3.87); p=0.20
Alcohol use					
No	59 (30.4)	135 (69.6)	Reference		-
Yes	21 (28.8)	52 (71.2)	0.92 (0.51-1.67)	0.801	-

Correlation between motivation to quit scores and nicotine dependence scores

Correlation analysis demonstrated a weak but statistically significant inverse relationship between Richmond motivation scores and nicotine dependence among both smokers (r=-0.182; p=0.015) and smokeless tobacco users (r=-0.174; p=0.032), indicating that higher nicotine dependence was associated with lower motivation to quit (Table 3).

**Table 3:** Correlation matrix showing the relationship between motivation to quit tobacco and nicotine dependence scores among tobacco users *p<0.05 **p<0.01

Variables	1	2	3
1. Richmond motivation to quit score	1.000	-	-
2. Smoking nicotine dependence score (FTND): smokers only	-0.182*	1.000	-
3. Smokeless tobacco nicotine dependence score (FTND-ST): smokeless-only users	-0.174*	0.479**	1.000

## Discussion

The present study assessed motivation to quit tobacco and its determinants among tobacco users attending a rural primary health center in Uttarakhand. Only 30% of participants demonstrated moderate-to-high motivation to quit tobacco, while the majority had low motivation. The duration of tobacco use, occupation, and nicotine dependence emerged as independent predictors of motivation to quit. Motivation to quit is a critical precursor to quit attempts and successful cessation and therefore represents an important target for tobacco control interventions [6,7].

The low level of motivation observed in the present study is consistent with the pattern reported earlier in Indian primary care and community settings, though our figure is at the lower end of the reported range. Panda et al., in a study of 1,569 tobacco users attending public health facilities in Andhra Pradesh and Gujarat, reported that only 12% of patients intended to quit within 30 days and 11% were ready to set a quit date [15]. In contrast, Dasgupta et al., in a community-based rural sample of 198 daily male smokers from Singur, West Bengal, reported that 76.3% of participants intended to quit smoking and 31.8% had attempted to quit in the past year [16]. Goyal et al., in a study of 425 tobacco users attending outpatient departments of a tertiary care hospital in Delhi, reported an overall willingness to quit of 70% [17]. Our estimate of 30% moderate-to-high motivation falls between the very low intention observed in the Andhra Pradesh/Gujarat public-facility sample and the higher figures observed in West Bengal and Delhi and likely reflects the specific characteristics of our rural North Indian PHC-based cohort, which included a substantial proportion of long-duration users (57.3% with >20 years of use) and older adults (46.1% aged ≥60 years).

A major finding of the present study was the strong inverse association between the duration of tobacco use and motivation to quit. Participants with 10-20 years and >20 years of tobacco use had 75% and 87% lower adjusted odds, respectively, of reporting moderate/high motivation compared with users of <10 years. A longer duration of use probably indicates a more established dependence, greater behavioral training, and repeated unsuccessful attempts to quit, all of which could lower confidence in effectively quitting. This finding is consistent with Goyal et al., who reported that tobacco users with ≤10 years of duration had significantly higher adjusted odds of willingness to quit compared with users of longer duration (AOR=1.8; 95% CI: 1.1-3.3) [17]. Additionally, it is consistent with the findings of Dasgupta et al., who found that a longer duration of smoking (≥25 years) was linked to a lower intention and attempt to quit in univariate analysis, even though this association did not reach independent significance in their multivariable model [16].

Nicotine dependence came out as another important determinant of motivation to quit. Participants with moderate-to-high nicotine dependence had significantly lower adjusted odds of moderate/high motivation (AOR=0.38; 95% CI: 0.19-0.77). This finding is biologically plausible because nicotine dependence creates both physiological and psychological barriers to cessation. It is strongly supported by the observations of Dasgupta et al., who reported that low nicotine dependence was an independent predictor of both intention to quit (AOR=2.98; 95% CI: 1.43-6.21) and attempt to quit (AOR=5.85; 95% CI: 2.85-12.00) among rural West Bengal smokers [16]. The significant negative correlation between Richmond motivation scores and both FTND (r=-0.182) and FTND-ST scores (r=-0.174) observed in the present study further supports the concept that increasing dependence reduces readiness and confidence to quit.

It should be noted, however, that the direction of the dependence-motivation relationship is not uniform across the international literature. de Granda-Orive et al., in a multicenter observational study of 314 smokers attending smoking cessation clinics in Spain and Argentina, found no significant association between nicotine dependence measured by the Fagerström Test for Cigarette Dependence (FTCD) and motivation to quit as assessed by the Richmond, Henri-Mondor Paris, Khimji-Watts, or visual analog scales, after adjustment for age and sex [18]. The authors attributed the absence of association partly to the selective nature of cessation-clinic populations and to methodological limitations of the motivation instruments themselves. In our primary care population, where the sample was not selected on cessation-seeking behavior, the inverse association between dependence and motivation was clearly demonstrable, suggesting that setting-related selection effects play an important role in shaping this relationship. Therefore, highly dependent tobacco users, particularly those identified at the primary care level, require more intensive behavioral counseling and pharmacological support than less dependent users.

Occupation was also significantly associated with motivation to quit in the adjusted model. Participants engaged in skilled or sales-related occupations demonstrated greater motivation to quit than unemployed individuals (AOR=2.66; 95% CI: 1.05-6.70). This may be explained by greater health awareness, increased social interaction, improved access to health information, and stronger workplace influences that encourage healthy behaviors. It is interesting to note that Dasgupta et al. found that, in their rural West Bengal cohort, agriculture was independently associated with a higher intention to quit (AOR=2.17; 95% CI: 1.01-4.63) [16]. This highlights the fact that while the specific occupational categories linked to higher motivation may differ by location and local economic structure, the overall pattern of occupation-related differences in cessation intent seems consistent.

Although age, educational status, socioeconomic status, age at the initiation of tobacco use, and diabetes mellitus showed significant associations in unadjusted analyses, these relationships did not remain significant after adjustment for confounding variables. This suggests that their effects may be mediated through dependence-related characteristics and the duration of tobacco use rather than exerting an independent influence on motivation. Panda et al. similarly observed that after adjustment, several sociodemographic variables lost their significance, while nicotine dependence and previous quit attempts remained the most robust predictors of intention to quit and setting a quit date [15].

The results of this study have significant ramifications for primary healthcare tobacco cessation services. Frequent screening using straightforward instruments such as the Richmond Scale and FTND/FTND-ST can help identify tobacco users with strong dependence and low motivation early on. Incorporating structured cessation counseling into PHCs and NP-NCD clinics may enhance rural tobacco users' preparedness to stop and boost the number of successful cessation efforts. Long-term users and highly dependent people should receive special attention because they seem to be the least willing to stop and hence need intense help.

Limitations

This study has some limitations worth noting. Being cross-sectional, it shows associations, not causation; the duration of tobacco use, nicotine dependence, and occupation were linked to motivation to quit, but we cannot say they cause it. Motivation also is not fixed; it shifts over time, and our one-time Richmond Scale reading captures only a single moment. Generalizability to tobacco users in the community is also limited by the PHC-based sample. The parent study was built to estimate nicotine dependence prevalence, not to explain motivation, and only 80 participants landed in the moderate/high-motivation group. With so few relative to the predictors studied, confidence intervals came out wide, as with the adjusted odds ratio of 0.13 (95% CI: 0.03-0.52) for long-term users. These findings should be read as hypothesis-generating, not conclusive. Recall and social desirability bias may have an impact on self-reported alcohol and smoke usage. Several factors that plausibly shape motivation, including self-efficacy, mental health, tobacco use among household members and peers, earlier contact with cessation services, and access to pharmacotherapy, were not recorded, leaving room for residual confounding.

Recommendations

At the level of routine primary care, both nicotine dependence and motivation to quit can be assessed quickly and at little cost using the FTND/FTND-ST and the Richmond Scale, and there is a good case for building these into the noncommunicable disease (NCD) clinic workflow at PHCs. Used together, they let frontline providers gauge where a tobacco user stands on both readiness and dependence and tailor the intervention to match.

The tobacco users who were least motivated in this cohort, long-duration users and those with moderate-to-high dependence, are precisely the ones who need more than brief advice. They should be prioritized for repeated behavioral counseling and, where appropriate, pharmacological support. Strengthening what already exists within the PHC and NP-NCD platforms, through follow-up contacts, clear referral pathways to more intensive services, and provider training, would help turn the modest motivation seen here into genuine quit attempts.

Based on the study findings, a stepwise PHC-level tobacco cessation model is recommended to strengthen routine screening, motivation assessment, tailored counseling, appropriate treatment, and regular follow-up at the primary healthcare level (Figure 3).

**Figure 3:**
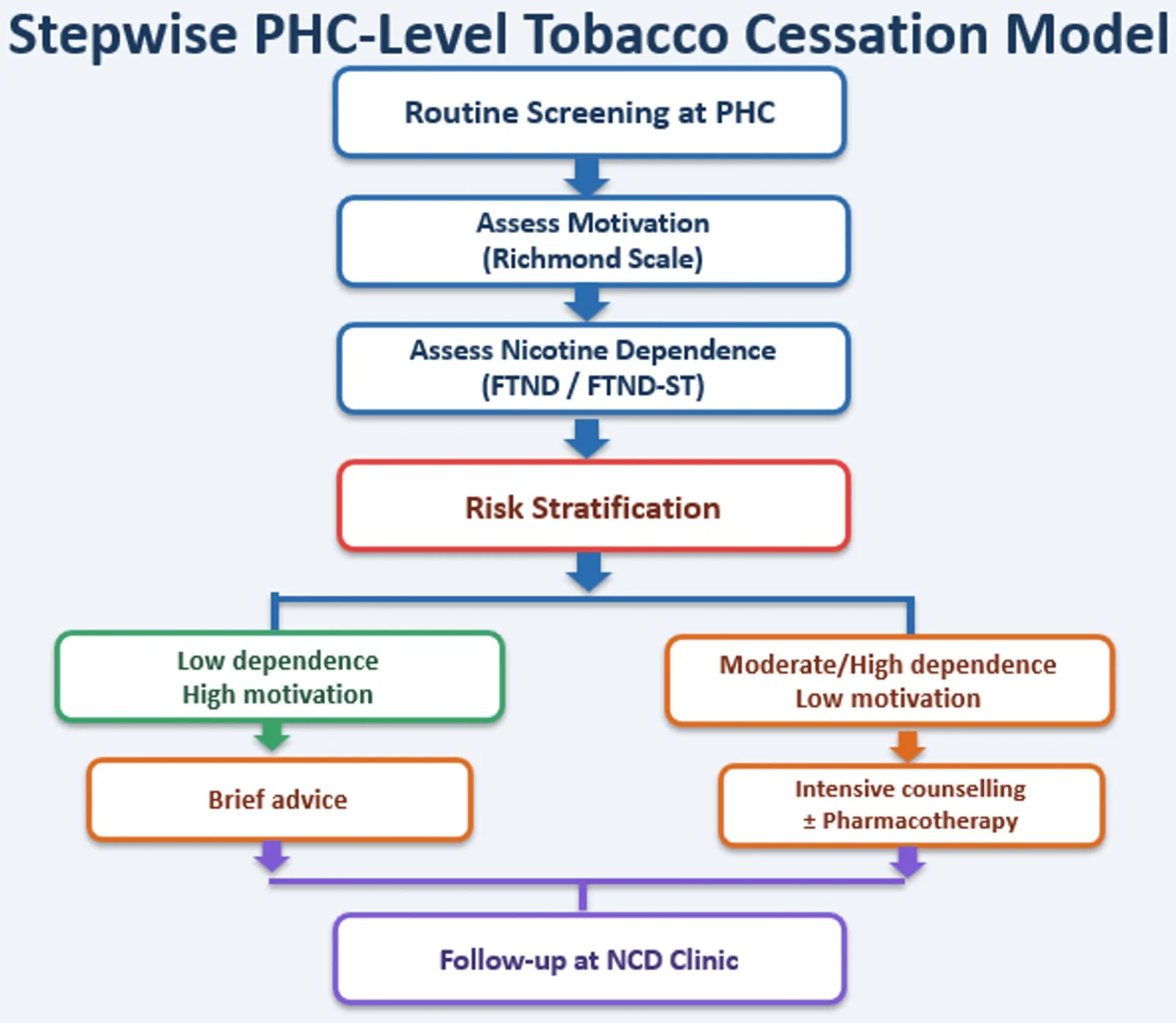
Stepwise PHC-level tobacco cessation model based on study findings PHC, primary health center; NCD, noncommunicable disease

Longitudinal work is needed to understand how motivation changes over time and whether interventions delivered early at the PHC level can raise it and carry it through to confirmed abstinence. Larger multicenter studies across varied rural and urban settings would also improve generalizability and make it possible to examine the psychosocial factors, self-efficacy, and prior quit experience among them that this analysis was unable to reach.

## Conclusions

Moderate-to-high motivation to quit tobacco was observed in only 30% of tobacco users attending the rural PHC. Longer duration of tobacco use and higher nicotine dependence were independently associated with lower motivation to quit, whereas participants engaged in skilled or sales occupations demonstrated higher motivation. The routine assessment of nicotine dependence and motivation using simple screening tools may help identify tobacco users requiring intensive cessation support at the primary healthcare level.
